# Characterization of Kunitz-Domain Anticoagulation Peptides Derived from *Acinetobacter baumannii* Exotoxin Protein F6W77

**DOI:** 10.3390/toxins16100450

**Published:** 2024-10-21

**Authors:** Fang Sun, Xiaolin Deng, Huanhuan Gao, Li Ding, Wen Zhu, Hongyi Luo, Xiangdong Ye, Xudong Luo, Zongyun Chen, Chenhu Qin

**Affiliations:** 1 Institute of Biomedicine, Hubei Key Laboratory of Embryonic Stem Cell Research, and Hubei Key Laboratory of Wudang Local Chinese Medicine Research, College of Basic Medicine, Hubei University of Medicine, Shiyan 442000, China; 2016202040055@whu.edu.cn (F.S.); lynn6444@163.com (X.D.); 15871101737@163.com (H.G.); dl2168@163.com (L.D.); zhuwen0712@126.com (W.Z.); 2016202040022@whu.edu.cn (H.L.); yexiangdong@hbmu.edu.cn (X.Y.); luoxudong@hbmu.edu.cn (X.L.); 2 Hubei Key Laboratory of Wudang Local Chinese Medicine Research, Hubei University of Medicine, Shiyan 442000, China; 3 Department of Clinical Laboratory, Dongfeng Hospital, Hubei University of Medicine, Shiyan 442000, China

**Keywords:** bacterial Kunitz-domain, anticoagulation peptide, coagulation factor XIa, bacterial exotoxin protein, intrinsic coagulation pathway

## Abstract

Recent studies have revealed that the coagulation system plays a role in mammalian innate defense by entrapping bacteria in clots and generating antibacterial peptides. So, it is very important for the survival of bacteria to defend against the host coagulation system, which suggests that bacterial exotoxins might be a new source of anticoagulants. In this study, we analyzed the genomic sequences of *Acinetobacter baumannii* and a new bacterial exotoxin protein, F6W77, with five Kunitz-domains, KABP1-5, was identified. Each Kunitz-type domain features a classical six-cysteine framework reticulated by three conserved disulfide bridges, which was obviously similar to animal Kunitz-domain peptides but different from plant Kunitz-domain peptides. Anticoagulation function evaluation showed that towards the intrinsic coagulation pathway, KABP1 and KABP5 had apparently inhibitory activity, KABP4 had weak inhibitory activity, and KBAP2 and KABP3 had no effect even at a high concentration of 20 μg/mL. All five Kunitz-domain peptides, KABP1-5, had no inhibitory activity towards the extrinsic coagulation pathway. Enzyme-inhibitor experiments showed that the high-activity anticoagulant peptide KABP1 had apparently inhibitory activity towards two key coagulation factors, Xa and XIa, which was further confirmed by pull-down experiments that showed that KABP1 can bind to coagulation factors Xa and XIa directly. Structure-function relationship analyses of five Kunitz-type domain peptides showed that the arginine of the P1 site of three new bacterial anticoagulants, KABP1, KABP4 and KABP5, might be the key residue for their anticoagulation activity. In conclusion, with bioinformatics analyses, peptide recombination, and functional evaluation, we firstly found bacterial-exotoxin-derived Kunitz-type serine protease inhibitors with selectively inhibiting activity towards intrinsic coagulation pathways, and highlighted a new interaction between pathogenic bacteria and the human coagulation system.

## 1. Introduction

The mammalian coagulation system was traditionally regarded as an essential functional network for blood hemostasis [1]. However, more and more evidences indicate that coagulation factors also contribute to the effective elimination of bacteria in mammals [2,3,4]. Recent studies have indicated that the coagulation system contributes to mammalian innate defense, and some coagulation factors can entrap bacteria inside clots and generate small antibacterial peptides [5]. So, it might be an important survival adaptation strategy for bacteria to defend against the host coagulation system, which suggests that bioactive substances secreted by bacteria might be a source of new anticoagulants.

The coagulation cascade is typically activated through two distinct pathways: one is the contact system (intrinsic pathway), and the other is the tissue factor (extrinsic pathway) system [6]. Microbe-specific activation of different coagulation pathways was suggested to occur via diverse microbial structure substances, including the clumping factor [7], coagulase [8], Efb [9], FnbpA [10], SSL10 from *Staphylococcus aureus* [11], SclA and SclB from Group A streptococci [12], and PolyP from Various bacterial species [13]. These activators might result in the formation of pro-inflammatory mediators and pro-coagulation factor activation and clotting [14]. Because microbial structure substances can activate host coagulation systems and cause microorganisms to be killed by the host’s coagulation system, bioactive substances secreted by bacteria might have anticoagulant activity to defend from the killing [1]. In fact, some anticoagulant bioactive substances have been found from pathogenic bacteria, such as a secreted, zinc-dependent, metallo-endopeptidase CpaA from *Acinetobacter baumannii* [15,16], and an extracellular serine protease EspP from Enterohemorrhagic *Escherichia coli* [17]. It might be a new strategy to discover new anticoagulants from bacterial exotoxin proteins. However, little work has been done in this new field.

Kunitz-type peptides are a kind of important serine protease inhibitor resource. Here, by comprehensive analyses of the genomic sequences of *Acinetobacter baumannii*, a new bacterial exotoxin protein, F6W77, with five Kunitz-domains was identified. Through a combination of bioinformatics analyses, peptide recombination, and functional evaluation, we firstly found bacterial derived Kunitz-domain anticoagulants and highlighted a new interaction between pathogenic bacteria and the human coagulation system.

## 2. Results

### 2.1. Sequence Analyses and Kunitz-Domain Identification of a New Bacterial Exotoxin Protein, F6W77, from Acinetobacter baumannii

**Figure 1:**
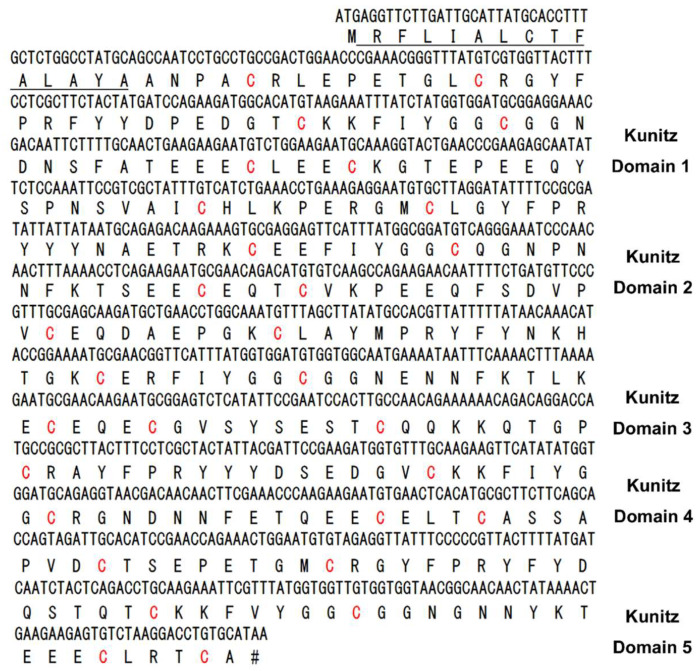
Precursor nucleotide sequence and deduced amino acid sequence of a secreted protein with multiple Kunitz-type domains from the bacteria of *Acinetobacter baumannii*. The predicted protein F6W77_19310 is shown below the nucleotide sequence of genomic DNA (KAB1093641.1, from the bacteria of *Acinetobacter baumannii*). The signal peptide is underlined. The mature protein contains 303 residues with five Kunitz-type domains, and each Kunitz-type domain possesses a classical six cysteine framework.

**Figure 2:**
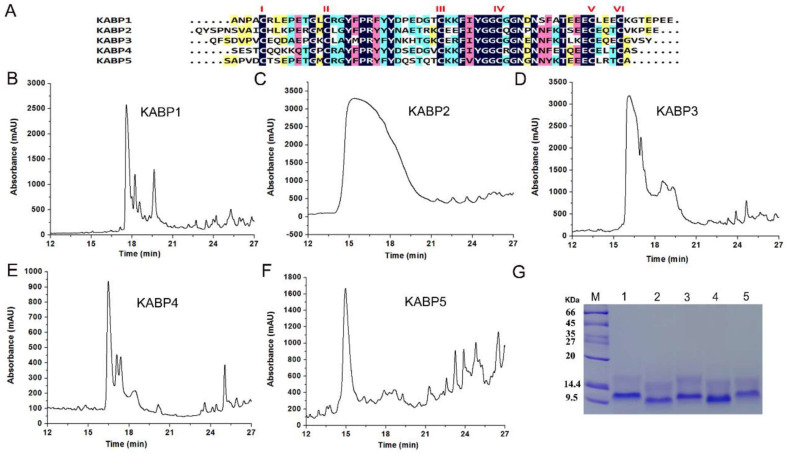
Expression and purification of five Kunitz-domain peptides, KABP1, KABP2, KABP3, KABP4 and KABP5, derived from the 303-aa secreted protein of the bacteria of *Acinetobacter baumannii*. (**A**). Primary structures of five Kunitz-domain peptides named KABP1(Kunitz-type *Acinetobacter baumannii* peptide 1), KABP2, KABP3, KABP4 and KABP5, and sequence alignments of five Kunitz-domain peptides; (**B**). Purification of the peptide KABP1 by RP-HPLC; (**C**). Purification of the peptide KABP2 by RP-HPLC; (**D**). Purification of the peptide KABP3 by RP-HPLC; (**E**). Purification of the peptide KABP4 by RP-HPLC; (**F**). Purification of the peptide KABP5 by RP-HPLC. (**G**). SDS-PAGE identification of five recombinant Kunitz-domain peptides.

Based on the previous work about Kunitz-type peptides from our and other laboratories, we found that peptides with Kunitz-type structural folds are effective molecular scaffolds [18,19,20,21,22,23,24,25,26,27,28]. *Acinetobacter baumannii* needs to defend against the host coagulation system, and might produce exotoxin proteins with anticoagulation activity. Using BLAST, we searched genomic and cDNA data of *Acinetobacter baumannii* from the NCBI website, and a new secreted exotoxin protein, F6W77, was identified. The exotoxin precursor codes for a 15-aa signal peptide and a 303-aa mature protein including five Kunitz-type domains, all of which possessed a classical six-cysteine framework reticulated by three conserved disulfide bridges (Figure 1 and Figure 2A), suggesting their potential function as defense against some serine proteases.

### 2.2. Expression of Kunitz-Domain Peptides from the Bacterial Exotoxin Protein F6W77 of Acinetobacter baumannii

Using the pET-28a expression and purification system that we had built before, five Kunitz-domain peptides were expressed and purified. All five peptides with enriched cysteine residues were found to exclusively accumulate in inclusion bodies, so they were further refolded in vitro with the classical GSH-GSSG system as we have described before [19,29]. The renatured protein was finally purified by high-performance liquid chromatography (HPLC) on a C18 column (10 × 250 mm^2^, 5 μm Dalian Elite, China). Peaks were detected at 230 nm. The fraction containing recombinant peptide was eluted at about 21 min, which was a single peak. The fraction was collected manually and lyophilized, which corresponded to the Kunitz-domain peptide (Figure 2B–F). SDS-PAGE identification showed the five recombinant Kunitz-domain peptides had similar molecular weights that were consistent with their similar residue numbers, which suggested that recombinant Kunitz-domain peptides were obtained successfully (Figure 2G).

### 2.3. Anticoagulation Activity Characterization of Five Kunitz-Domain Peptides from the Bacterial Exotoxin Protein F6W77

**Figure 3:**
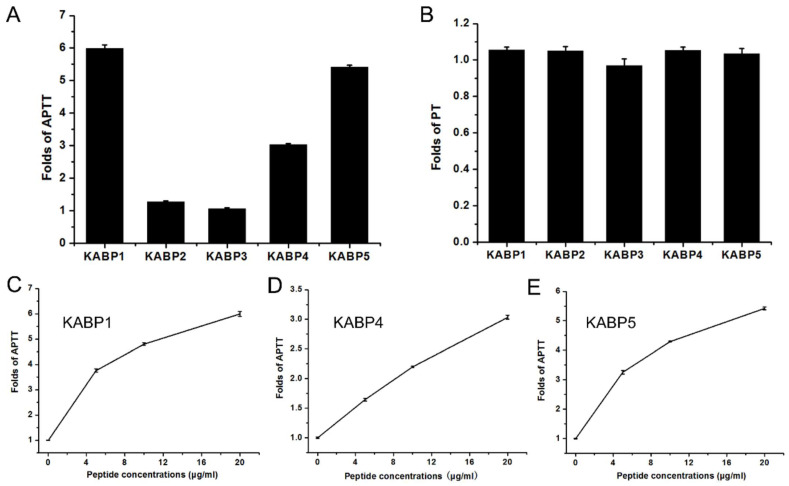
Kunitz-domain peptides KABP1, KABP4 and KABP5 are new anticoagulants from the bacteria of *Acinetobacter baumannii* that inhibit the intrinsic coagulation pathway selectively. (**A**). Anticoagulant activities of five Kunitz-domain peptides with the same concentration of 20 μg/mL using the APTT test; (**B**). Anticoagulant activities of five Kunitz-domain peptides with the same concentration of 20 μg/mL using the PT test; (**C**). Anticoagulant activities of the peptide KABP1 with different concentrations evaluated by the APTT test; (**D**). Anticoagulant activities of the peptide KABP4 with different concentrations evaluated by the APTT test; (**E**). Anticoagulant activities of the peptide KABP5 with different concentrations evaluated by the APTT test.

Then, APTT and PT tests were performed to detect activities of peptides on the intrinsic and extrinsic coagulation pathways, respectively. Our results showed that all five peptides had no activities towards the extrinsic coagulation pathway, but three peptides, KABP1, KABP4, and KABP5, had selective inhibitory activity towards the intrinsic coagulation pathway (Figure 3). Kunitz-domain peptides KABP1 and KABP5 prolonged APTT over the normal time period, and there was about a two-fold increase with about 2000 nM concentration (Figure 3). Together, these results showed recombinant peptides KABP1, KABP4, and KABP5 had selective and potent activity towards the intrinsic coagulation pathway, and two Kunitz-domain peptides, KABP2 and KABP3, had no anticoagulation towards both the intrinsic and extrinsic coagulation pathways. In summary, with the APTT test performed for detecting activities of peptides on the intrinsic coagulation pathway, and the PT test performed for detecting activities of peptides on the extrinsic coagulation pathway, three new anticoagulants (KABP1, KABP4, and KABP5) derived from the bacterial exotoxin protein F6W77 were discovered, which selectively inhibited the intrinsic coagulation pathway.

### 2.4. Two Kunitz-Domain Peptides, KABP1 and KABP5, Are Potent Inhibitors towards Coagulation Factors Xa and XIa

**Figure 4:**
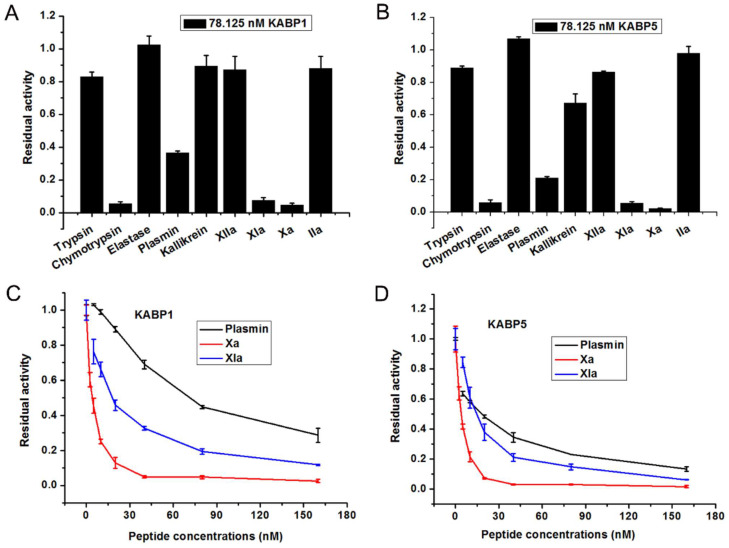
Serine protease inhibitory activities of KABP1 and KABP5 from the bacteria of *Acinetobacter baumannii*. (**A**). Representative serine protease inhibitory activity screening of the peptide KABP1 with the same concentration of 78.125 nM. Each column represents three independent experiments. (**B**). Representative serine protease inhibitory activity screening of the peptide KABP5 with the same concentration of 78.125 nM; (**C**). Serine protease inhibitory activity of the peptide KABP1 with different concentrations of 156.25 nM, 78.125 nM, 39.063 nM, 19.531 nM, 9.766 nM, 4.883 nM, 2.441 nM and the control; (**D**). Serine protease inhibitory activity of the peptide KABP5 with the concentrations as before. Each experiment was done at least three times.

Enzyme kinetics experiments further showed that recombinant peptides KABP1 and KABP5 are potent inhibitors towards coagulation factors XIa and Xa inhibitors (Figure 4), but had a weaker activity towards plasmin. Among the two Kunitz-domain peptides KABP1 and KABP5, the selectively inhibiting activity of KABP1 is good, so the kinetic mechanism and binding effect of KABP1 were further evaluated.

**Figure 5:**
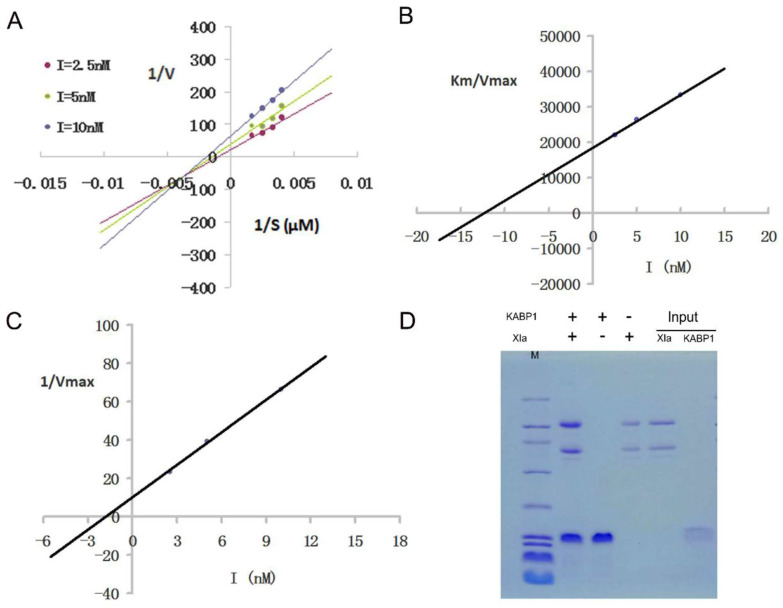
Kinetic mechanism of one Kunitz-domain peptide, KABP1, towards XIa. (**A**) The kinetic mechanism of KABP1 inhibiting coagulation factor XIa, which indicated that KABP1 was a mixed inhibitor of FXIa; (**B**) The slopes (Km/Vmax) of the primary Lineweaver-Burk graphs were plotted against the concentration of inhibitor. The inhibitory constant (Ki) is calculated from the intercept point on the *x*-axis; (**C**) The Y-intercept of the primary Lineweaver-Burk graphs were plotted against the concentration of inhibitor. The inhibitory constant (Ki′) is calculated from the intercept point on the *x*-axis; (**D**) The His-pull experiment showed that the anticoagulant KABP1 from the bacteria of *Acinetobacter baumannii* binds to the enzyme XIa directly. The Ki value of the inhibitor to the enzyme XIa is 12.4 nM, and the Ki′ value of the inhibitor to the XIa enzyme-substrate complex is 1.8 nM.

The kinetic mechanism experiment of the peptide KABP1 towards coagulation factor XIa showed that KABP1 was a unique mix-competitive XIa inhibitor that can bind to both enzymes and enzyme-substrate complexes (Figure 5). The Ki value that demonstrates the binding ability of the inhibitor to the enzyme XIa is 12.4 nM, and the Ki′ value that demonstrates the binding ability of the inhibitor to the XIa enzyme-substrate complex is 1.8 nM, which means the XIa inhibitor KABP1 has better binding ability to enzyme-substrate complexes than enzymes. Besides this, it also indicated that KABP1 was not a classical competitive inhibitor that binds to the active center of the enzyme coagulation XIa, but might have a novel binding site besides the active center.

**Figure 6:**
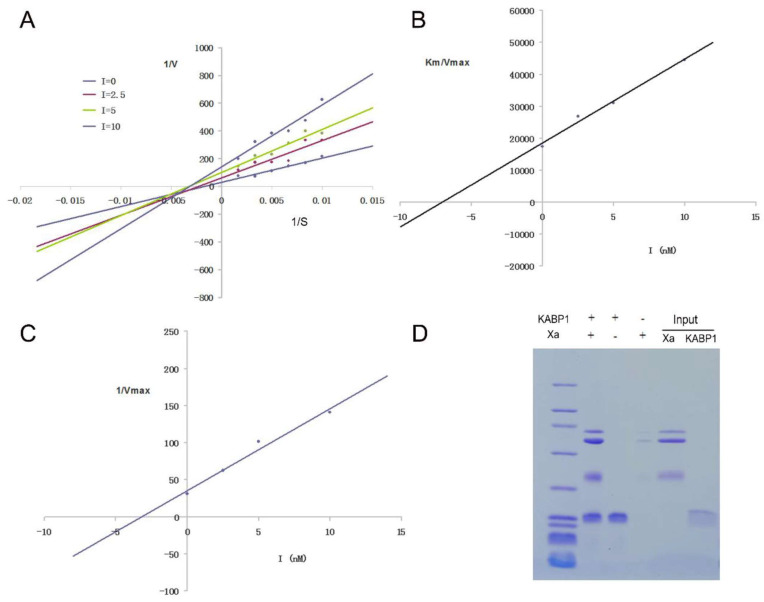
Kinetic mechanism of the peptide KABP1 towards Xa. (**A**). The kinetic mechanism of KABP1 inhibiting coagulation factor Xa, which indicated that KABP1 was a mixed inhibitor of FXa; (**B**). The slopes (Km/Vmax) of the primary Lineweaver-Burk graphs were plotted against the concentration of inhibitor; (**C**). The Y-intercept of the primary Lineweaver-Burk graphs were plotted against the concentration of inhibitor; (**D**). The His-pull experiment showed that theanticoagulant KABP1 from the bacteria of *Acinetobacter baumannii* binds to coagulation factor Xa directly. The Ki value of the inhibitor KABP1 to the enzyme Xa is 7.1 nM, and the Ki′ value of the inhibitor to the Xa enzyme-substrate complex is 3.2 nM.

The kinetic mechanism experiment of the inhibitor KABP1 towards another enzyme coagulation, Xa, was done with similar methods, and the results showed that KABP1 also was a mixed Xa inhibitor with both competitive and uncompetitive inhibitory mechanisms, which was consistent with the inhibiting mechanism of the inhibitor towards the enzyme coagulation factor XIa (Figure 6). The Ki value of the inhibitor KABP1 to the enzyme Xa is 7.1 nM, and the Ki′ value of the inhibitor KABP1 to the Xa enzyme-substrate complex is 3.2 nM, which means the inhibitor KABP1 also has better binding ability to enzyme-substrate complexes than enzymes towards coagulation factor Xa. His-pull-down experiments further showed that the inhibitor KABP1 can bind to the two key enzymes, XIa and Xa, directly (Figure 5D and Figure 6D), which was consistent with the enzyme and inhibitor reaction kinetics experiments.

### 2.5. Structure-Activity Relationship Comparison of Five Kunitz-Domain Peptides

**Figure 7:**
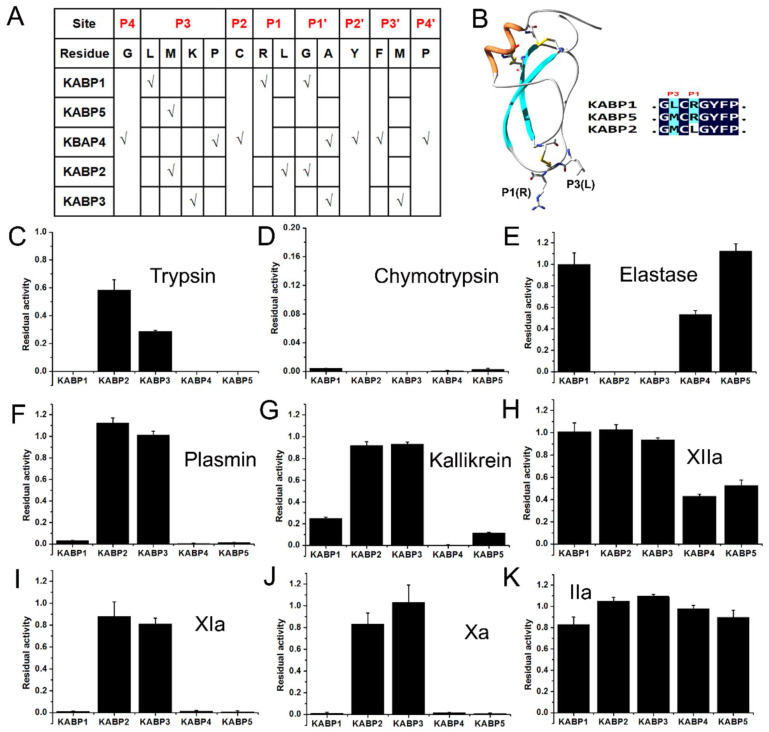
Structure and activity relationship of KABP1, the first bacteria-derived Kunitz-domain peptide anticoagulant. (**A**). Sequence analyses of KABP1 at the potential functional sites P4, P3, P2, P1, P1′, P2′, P3′ and P4′; (**B**). The P1-site arginine, but not P3′ site of KABP1, might be the key site for its anticoagulation activity; (**C**). Inhibitory activity of five Kunitz-domain peptides towards trypsin; (**D**). Inhibitory activity of five Kunitz-domain peptides towards chymotrypsin; (**E**). Inhibitory activity of five Kunitz-domain peptides towards elastase; (**F**). Inhibitory activity of five Kunitz-domain peptides towards plasmin; (**G**). Inhibitory activity of five Kunitz-domain peptides towards kallikrein; (**H**). Inhibitory activity of five Kunitz-domain peptides towards XIIa; (**I**). Inhibitory activity of five Kunitz-domain peptides towards XIa; (**J**). Inhibitory activity of five Kunitz-domain peptides towards Xa; (**K**). Inhibitory activity of five Kunitz-domain peptides towards IIa.

All five Kunitz-domain peptides are the classical Kunitz-type peptide, and adopt a six-cysteine framework reticulated by three conserved disulfide bridges. Sequential and structural alignments showed that the functional loop of the classical Kunitz-type peptide was major from P4 site to P4′ site [30,31], which corresponded to the sequence “G12-L13-C14-R15-G16-Y17-F18-P19” in the KABP1 peptide. Based on these analyses, the structure-activity relationship comparison of five Kunitz-domain peptides, KABP1, KABP2, KABP3, KABP4, and KABP5, was further studied (Figure 7). For the P1 site, there are two kinds of amino acids: one is Arg (R) for peptides KABP1, KABP4, and KABP5, and the other is Leu (L) for peptides KABP2 and KABP3. In combination with the anticoagulation activities of the five Kunitz-domain peptides, it can be found that the base amino acid residue in the P1 site might be the key functional residue for their intrinsic coagulation pathway inhibiting activities. It also means that the key residues of three anticoagulants, KABP1, KABP4, and KABP5, might be their P1 sites’ arginine residue (R), which is R15 for KABP1 and KABP4, and R16 for KABP5.

Interestingly, KABP2 and KABP3 had good elastase inhibiting activity, but KABP1, KABP4, and KABP5 had no inhibiting activity towards elastase at the same concentration. This suggested that the hydrophobic amino acid Leu might be the key functional residue for elastase inhibiting, which further indicated that the P1 site might have the key residue for the anticoagulation of Kunitz-domain peptides KABP1, KABP4, and KABP5. For KABP1, KABP4, and KABP5, we found that KABP4 and KABP5 had weak coagulation factor XIIa-inhibiting activity, but KABP1 had no activity towards XIIa. Considering that P4, P2, P1, P2′, P3′, and P4′ of three Kunitz-domain peptides KABP1, KABP4, and KABP5 are the same, two functional sites, P3 and P1′, might be associated with their different inhibiting activity towards coagulation factor XIIa. In addition, the P1′ site of KABP1 and KABP5 is the same (G), but is different from the P1′ site of KABP4, which suggested that the P3 site might the most important functional site for the XIIa-inhibiting activity of Kunitz-domain peptides KABP4 and KABP5. In conclusion, the P1-site arginine of KABP1, KABP4, and KABP5 might be associated with their common anticoagulation activity, and the P3 site might also influence their activities (Figure 7B). In addition, the P1-site leucine of KABP2 and KABP3 might be associated with their common elastase-inhibiting activity [32].

## 3. Discussion

The intrinsic coagulation pathway and the extrinsic coagulation pathway are two basic processes to initiate blood clotting. The former is started by the XIIa-kallikrein mutual activation system, which is initiated by collagen and other negatively-charged molecules, such as some compounds from pathogenic bacteria. After that, FXIIa converts FXI into FXIa, and Factor XIa activates FIX [33,34,35,36]. At last, the tenase complex activates FX to FXa to start the common blood clotting [37,38]. Recent studies indicated that interference with the intrinsic coagulation pathway may not cause a significant bleeding risk [37,39]. Here, we firstly reported three bacterial anticoagulants, KABP1, KABP4, and KABP5, acting towards the intrinsic coagulation pathway, which suggested that pathogenic exotoxin proteins might be a new resource for novel anticoagulants to fight against thrombotic related diseases.

**Figure 8:**
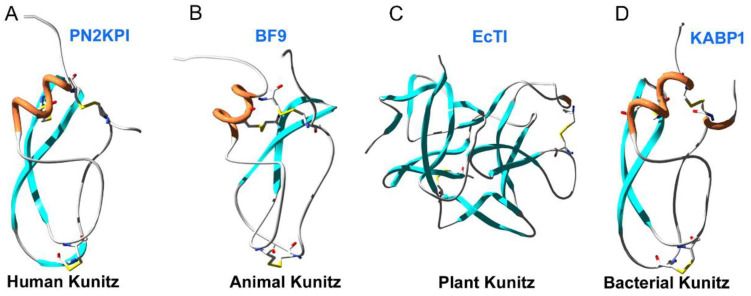
The 3-D structure of the Kunitz-domain peptide of bacteria is similar to human and animals, but different from plants. (**A**). Structure of a human Kunitz-domain peptide PN2KPI (PDB code: 1ZJD); (**B**). Structure of an animal Kunitz-domain peptide BF9 (PDB code: 1JC6); (**C**). Structure of a plant Kunitz-domain peptide EnTI (PDB code: 4J2K); (**D**). Structural modelling of the first bacterial Kunitz-domain peptide, KABP1, by a Swiss-model sever with Boophilin (PDB code: 2ODY) as a template.

Kunitz-type bioactive peptides are important serine protease inhibitor resources and many Kunitz-type anticoagulation peptides have been found in previous work, such as PN2KPI from human [25], Amblyomin-X from tick [27], RVV inhibitor II from snake [40], BF9 from snake [29,41], Fasxiator from snake [41,42], Simukunin from fly [43], joannsin from millipede [44], KPI from Limulidae [45], Bicolin from snake and TAP from blood-sucking animals [45,46,47], *En*KT1 from Fluke [48], SjKI-1, Schixator and SmKI-1 from parasite worm schistosoma [22,49,50]. Bacteria derived Kunitz-domain anticoagulants were found firstly in our present work (Table 1), which was similar to the Kunitz-type peptides of animals, but different from the Kunitz-type peptides of plants completely [51] (Figure 8). Because almost all animals have Kunitz-domain peptides to defend against diverse serine proteases and few Kunitz-domain peptides have been found from bacteria [52,53,54], our present work provided an interesting idea that bacteria-derived Kunitz-domain peptides might be transferred from host animals, but more evidence is needed to confirm this idea in the future.

During long-time interactions with the host hemostatic system, bacteria have evolved several bioactive compounds to inhibit host blood coagulation, such as a secreted, zinc-dependent, metallo-endopeptidase CpaA from *Acinetobacter baumannii*, and an extracellular Serine Protease EspP from Enterohemorrhagic *Escherichia coli* [15,16,17]. As we know, both CpaA and EspP are serine proteases that can modulate the host coagulation system. Our present work showed that serine protease inhibitors such as Kunitz-type inhibitors might also be present in bacterial secreted exotoxin proteins that also can modulate the host coagulation system [55]. The discovery of bacterial-exotoxin-derived Kunitz-domain anticoagulants highlights a new interaction between pathogenic bacteria and human coagulation system. Besides this, bacterial exotoxin proteins might be a new source of anticoagulants, especially for the intrinsic coagulation pathway.

In summary, by comprehensive analyses of the genomic sequences of *Acinetobacter baumannii*, a new bacterial exotoxin protein, F6W77, with five Kunitz-domains was identified. By combining peptide recombination with functional evaluation, we firstly found three new bacterial-exotoxin-derived Kunitz-domain anticoagulants, KABP1, KABP4, and KABP5, and highlighted a new interaction between pathogenic bacteria and the human coagulation system.

## 4. Materials and Methods

### 4.1. Bioinformatic Analysis of a Bacterial Exotoxin Protein, F6W77_19310, from the Bacteria Acinetobacter baumannii

Amino acid sequences of open reading frames were identified using DNAMAN. After excluding signal peptides from the open reading frame sequence, the sequence similarity was further analyzed by searching against the GenBank NCBI database (http://www.ncbi.nlm.nih.gov/blast, accessed on 12 September 2024) using BLAST algorithms. The atomic structure of all Kunitz-domain peptides was modeled in the SWISS-MODEL server (https://www.swissmodel.expasy.org, accessed on 13 September 2024).

### 4.2. Recombinant Plasmid Construction of Kunitz-Domain Peptides

Recombinant plasmids pET-28a-KABP1, pET-28a-KABP2, pET-28a-KABP3, pET-28a-KABP4, and pET-28a-KABP5 were constructed by overlapping PCR in our group according to our previous work [56,57]. All recombinant plasmids were verified by DNA sequencing and transformed into the competent cell *Escherichia coli* BL21 (DE3) before expression.

### 4.3. Recombinant Expression and Purification of Kunitz-Domain Peptides

All Kunitz-domain peptides were expressed and purified by a similar expression system as we have described before [56,57]. For example, the plasmid of pET-28a-KABP1 was transformed into competent Escherichia coli BL21 (DE3) cells for expression. The recombinant KABP1 was found to exclusively accumulate in inclusion bodies, so KABP1 was further refolded in vitro as we have described before [19,29].

### 4.4. Activated Partial Thromboplastin Time (APTT) and Prothrombin Time (PT)

APTT and PT experiments were used to evaluate the biological activities of Kunitz-domain peptides towards the intrinsic coagulation pathway and the extrinsic coagulation pathway as we have described before. For the APTT experiment, the reaction system is 0.1 mL human plasma, 0.1 mL APTT reagent (md-pacific, Tianjin, China), and 0.1 mL 25 mM CaCl2. For the PT experiment, 0.05 mL peptide with different concentrations was added to 0.1 mL plasma and incubated for 10 min, and clotting was initiated by the addition of 0.2 mL thromboplastin to the mixture.

### 4.5. Coagulation Factors Inhibitory Activity Assay

The inhibitory activity of KABP1 and other peptides towards coagulation factors were tested as before [19,41,57]. The inhibitory activity of KABP1 towards plasmin was tested by the following system: the serine protease plasmin (20 nM) and the substrate S2251 (1.0 mM). Inhibitory tests for trypsin, chymotrypsin, and elastase were also carried out in the same manner as we have described before [19]. The inhibitory results are showed as means ± SD.

## Figures and Tables

**Table 1:** Natural Kunitz-type anticoagulant peptides from different organisms.

Peptide	Source	P2	P1	P1′	P2′	Target	Ref
KABP1	Bacteria	C	R	G	Y	FXIa, FXa	This work
PN2KPI	Human	C	R	A	M	FXIa, FIXa, FXa, Kallikrein	[25]
RVV inhibitor II	Snake	C	R	G	H	Kallikrein	[40]
Simukunin	Fly	C	R	A	L	FXa, FIXa, FXIa	[43]
*En*KT1	Fluke	C	R	A	S	FXa, Kallikrein	[48]
SjKI-1	Fluke	C	R	A	S	FXa, Kallikrein	[22]
Schixator	Fluke	C	R	G	Y	FXa and FXIa	[50]
SmKI-1	Fluke	C	R	A	L	FXa, Kallikrein	[49]
joannsin	Millipede	C	R	A	R	FXa	[44]
KPI	Limulidae	C	R	A	G	Kallikrein	[45]
BF9	Snake	C	N	A	L	FXIa	[29]
Fasxiator	Snake	C	N	A	L	FXIa, FXa	[42]
Amblyomin-X	Tick	C	S	N	K	FXa	[27]
TAP	Tick	C	D	S	N	FXa	[46]
Bicolin	Wasp	C	Q	S	S	IIa	[47]

## Data Availability

All data supporting the results can be found within the manuscript.
